# To Target or Not to Target: Active vs. Passive Tumor Homing of Filamentous Nanoparticles Based on *Potato virus X*

**DOI:** 10.1007/s12195-015-0388-5

**Published:** 2015-04-08

**Authors:** Sourabh Shukla, Nicholas A. DiFranco, Amy M. Wen, Ulrich Commandeur, Nicole F. Steinmetz

**Affiliations:** Department of Biomedical Engineering, Case Western Reserve University Schools of Medicine and Engineering, Cleveland, OH 44106 USA; Institute for Molecular Biotechnology, RWTH Aachen University, Worringer Weg 1, 52074 Aachen, Germany; Department of Macromolecular Science and Engineering, Case Western Reserve University Schools of Medicine and Engineering, Cleveland, OH 44106 USA; Department of Radiology, Case Western Reserve University Schools of Medicine and Engineering, Cleveland, OH 44106 USA; Department of Materials Science and Engineering, Case Western Reserve University Schools of Medicine and Engineering, Cleveland, OH 44106 USA

**Keywords:** Nanoparticle shape, Cancer, Tumor targeting, Biodistribution, Integrins

## Abstract

Nanoparticles are promising platforms for the diagnosis and treatment of cancer. Diverse classes and shapes of materials have been investigated to establish design principles that achieve the effective partitioning of medical cargos between tumors and healthy tissues. Molecular targeting strategies combined with specific nanoparticle shapes confer tissue-specificity on the carriers, allowing the cell-specific delivery of cargos. We recently developed a filamentous platform technology in which the plant virus *Potato virus X* (PVX) was used as a scaffold. These particles are flexible 515 × 13 nm filaments that encourage passive tumor homing. Here we sought to advance the PVX platform by including a molecular targeting strategy based on cyclic RGD peptides, which specifically bind to integrins upregulated on tumor cells, neovasculature, and metastatic sites. Although the RGD-targeted filaments outperformed the PEGylated stealth filaments *in vitro*, enhanced tumor cell targeting did not translate into improved tumor homing *in vivo* in mouse tumor models. The RGD-PVX and PEG-PVX filaments showed contrasting biodistribution profiles. Both formulations were cleared by the liver and spleen, but only the stealth filaments accumulated in tumors, whereas the RGD-targeted filaments were sequestered in the lungs. These results provide insight into the design principles for virus-based nanoparticles that promote the delivery of medical cargos to the appropriate cell types.

## Introduction

Cancer is the leading cause of death in the developed world and is also prevalent in the developing world. To improve survival and the quality of life, more efficacious therapies are required, especially to treat patients with tumors that resist conventional chemotherapy. Nanoparticles equipped with targeting ligands and medical cargo are promising approaches for cancer diagnosis and therapy because nanoparticle-based formulations enable the tissue-specific delivery of contrast agents for molecular imaging and/or toxic payloads for therapeutic intervention.^17^

**Figure 1:**
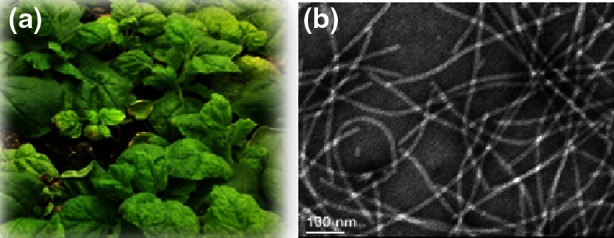
(**a**) *Nicotiana benthamiana* plants infected with *Potato virus X* (PVX) and (**b**) a negatively-stained transmission electron micrograph of PVX.

There is mounting evidence that elongated, filamentous nanomaterials are advantageous for drug delivery. Non-spherical materials show increased margination toward the vessel wall, which improves the efficiency of tumor homing.^6^^–^8^,^^10^^,^^14^^,^^15^^,^^18^ Elongated materials present ligands more effectively to target cells and the flat vessel wall than their spherical counterparts.^12^^,^^18^^,^^38^ Furthermore, elongated materials are more likely to resist immune detection and macrophage uptake, thus contributing to synergistic target enhancement.^3^^,^^39^ Nevertheless, most platform technologies currently under development are spherical or elongated low-aspect-ratio materials. Filomicelles mimic the shape of filamentous viruses and are therefore exceptions, but these are micron-sized and therefore technically they are not nanomaterials.^14^ The synthesis of high-aspect-ratio materials remains challenging because it is impossible to avoid polydispersity and the combination of synthetic chemistry and nanotechnology cannot yet mimic what nature has achieved, i.e., self-assembly and programmability at the atomic level. Therefore, we have developed a bioinspired platform technology based on filamentous plant viruses, specifically *Potato virus X* (PVX), which is shown in Fig. 1.

The PVX capsid is a flexible 515 × 13 nm protein-based filament that consists of 1270 identical copies of a coat protein unit suitable for genetic and chemical modification.^19^ The flexible nature of the material is advantageous because flexible nanomaterials can pass through restrictions and penetrate tissue more easily.^20^ We have shown that PVX-based filaments achieve more efficient partitioning between tumor and liver tissue compared to spherical nanoparticles.^31^ The shape-mediated enhanced tumor homing is reproducible in a variety of models, including human tumor xenografts of breast cancer, fibrosarcoma, squamous sarcoma and brain and colon cancers.^31^^,^^32^

A combination of prolonged circulation and the enhanced permeability and retention (EPR) effect are thought to contribute to passive tumor homing and accumulation.^17^ This phenomenon has been observed in many nanoparticle systems, including clinical formulations of Doxil^®^ (a liposomal formulation of doxorubicin) and Abraxane^®^ (an albumin nanoparticle formulation carrying paclitaxel). Both formulations increase the efficacy of their payloads, reflecting their beneficial physiochemical properties and the pathophysiological characteristics of the target tissue. Nevertheless, there is some ambiguity and controversy surrounding the EPR-mediated tumor homing of nanoparticles, especially in clinical applications.^17^ Therefore, nanoparticles have been modified to include targeting ligands that interact specifically with targets such as growth factor receptors,^29^^,^^40^ tumor matrix proteins,^24^^,^^37^ signatures of the tumor-associated endothelium^4^^,^^36^ or combinations thereof.

The overexpression of *α*_v_*β*_3_, *α*_v_*β*_5_ and *α*_5_*β*_1_ integrins on cancer cells, neovasculature and metastatic sites has been confirmed in many human malignancies, and changes in integrin expression and function are associated with disease progression.^26^^,^^27^^,^^36^ Integrins are validated targets for RGD peptide ligands and several nanoparticle systems carrying these peptides are under development. Here we describe the development and testing of integrin-targeted PVX nanofilaments modified with cyclic RGD peptides. The specificity of RGD-targeted PVX was compared to the PEG-PVX stealth formulation using a combination of *in vitro* and *in vivo* testing.

## Materials and Methods

### PVX Propagation and Synthesis

PVX was produced through farming in *N. benthamiana* plants using previously established protocols^19^ and extracted at yields of 20 mg of pure PVX from 100 g of infected leaf material. RGD-PVX particles were synthesized using a two-step protocol. First, PVX was covalently modified with Alexa Fluor 647 (A647) succinimdyl ester (NHS-A647, Life Technologies) and the bifunctional linker succinimidyl-[(*N*-maleimidopropionamido)-tetraethyleneglycol] ester [SM(PEG)_4_, Pierce] targeting surface-exposed lysine residues on the viral coat proteins (PVX consists of 1270 identical copies of a coat protein, each of which has an exposed lysine side chain). Subsequently, cyclic RGD peptide with cysteine functionality (cyclo(Arg-Gly-Asp-d-Phe-Cys (cRGDfC), Peptides International) was covalently attached to PVX through Michael addition targeting the terminal maleimide group of the bifunctional linker. Specifically, PVX particles at a concentration of 2 mg mL^−1^ in 0.1 M potassium phosphate buffer (pH 7.0) were incubated for 2 h at room temperature with 5000 molar excess of NHS-A647 and 10,000 molar excess of SM(PEG)_4_ in the presence of DMSO [at a final concentration of 10% (v/v)]. PVX formulations were then purified using 10-kDa cut-off centrifugal devices (Millipore) to separate unreacted dyes and linker molecules. Then, PVX rods were reacted overnight with 10,000 molar excess of cRGDfC in 0.1 M potassium phosphate buffer (pH 7.0) and purified as described above.

PEG-PVX stealth filaments were synthesized by reacting PVX (at 2 mg mL^−1^ in 0.1 M potassium phosphate buffer pH 7.0) with 5000 molar excesses of NHS-PEG5000 (NanoCS) and NHS-A647 (Life Technologies) overnight using a one-pot synthesis protocol. A647-labeled PEG-PVX formulations were then purified using centrifugal filters as described above. All formulations were resuspended in 0.1 M potassium phosphate buffer (pH 7.0) and stored at 4 °C until further processing.

### UV/Visible Spectroscopy

UV/visible spectroscopy was performed using NanoDrop instrument to quantify labeling efficiency with fluorophores. The number of A647 labels per PVX was calculated based on Beer-Lambert law using the PVX and A647-specific extinction coefficients: *ε*_PVX_ = 2.97 mL mg^−1^ cm^−1^ at 260 nm, *ε*_A647_ = 270,000 M^−1^ cm^−1^ at 650 nm.

### Denaturing Gel Electrophoresis

SDS gel electrophoresis was carried out to analyze conjugation of RGD peptide and PEG chains to individual coat proteins. 10 *μ*g denatured protein samples were analyzed on 4–12% NuPage gels (Life Technologies) in 1× MOPS SDS running buffer (Life Technologies). Protein bands were visualized under white light after staining the gels with SimplyBlue SafeStain (Life Technologies). Protein bands were analyzed using ImageJ software (http://imagej.nih.gov).

### TEM

Diluted samples of PVX, RGD-PVX and PEG-PVX particles (20 *μ*L, 0.1 mg mL^−1^) were negatively stained with of 2% (w/v) uranyl acetate for 2 min on a carbon-coated copper grid. Samples were analyzed using a Zeiss Libra 200FE transmission electron microscope operated at 200 kV.

### Tumor Homing and Imaging Using Mouse Xenograft Models

All animal studies were carried out using IACUC-approved procedures. Human colon cancer xenografts were developed using HT-29 cells (ATCC) maintained in McCoy’s medium (Life Technologies) supplemented with 10% (v/v) fetal bovine serum (FBS), 1% (w/v) penicillin/streptomycin and 1% (w/v) glutamine (all from Life Technologies). Six-week-old male NCr-*nu/nu* nude mice maintained on an alfalfa-free diet (Teklad 2018S, Harlan Laboratories) were subcutaneously injected in the right flank with 2.5 × 10^6^ cells in 50 *μ*L medium mixed with an equal volume of matrigel (BD Matrigel, BD Biosciences, USA) using a microliter Hamilton 22 gauge syringe. One tumor was induced in each mouse. The animals were monitored daily and tumor homing studies commenced when tumors reached an average volume of 20–30 mm^3^ (typically within 12 days after the injection of HT-29 cells). Animals were assigned randomly into groups with *n* = 3 mice per formulation. PBS, PEG-PVX and RGD-PVX particles were administered intravenously *via* tail vein injection. At specific time points post-administration, mice were euthanized and major tissues (liver, lungs, kidney, heart, spleen, brain and tumors) were harvested and imaged in the Maestro™ fluorescence imager using combinations of yellow excitation/emission filters for A647 (excitation filter: 575–605 nm; and emission filter: 645 nm longpass). Region of interest (ROI) analysis was performed using the Maestro™ software to determine the fluorescence intensity from the respective tissues as compared to tissues from PBS injected mice. After imaging, tissues were frozen in OCT media for cryo-sectioning.

### Immunofluorescence

Intratumoral localization of RGD-PVX and PEG-PVX particles with respect to integrins (*α*_v_), macrophages (CD68) and endothelium (CD31) was determined using immunofluorescence analysis of 10 *μ*m thick tumors sections prepared from frozen tissues embedded in OCT medium (Fisher) using a Leica CM1850 cryostat. Tumor sections were fixed in 95% (v/v) ethanol for 20 min on ice and rinsed with cold PBS. Tumor sections were permeablized using phosphate buffered saline containing 0.02% (v/v) Tween-20 (PBST) twice for 10 min each and then blocked with 10% (v/v) goat serum (GS) in PBST for 60 min. Sections were then stained using a rabbit anti-integrin *α*_v_ antibody (1:500, Millipore) or rat anti-mouse CD68 (1:250) antibody (Biolegend) with 1% (v/v) GS in PBST for 60 min at room temperature. After washing thoroughly with PBST, tumor sections were incubated with A488-conjugated goat anti-rabbit secondary antibody (1:2000, Life Technologies) and A555-conjugated goat anti-rat antibody (1:1000, Life Technologies) with 1% (v/v) GS in PBST for 60 min. Slides were then washed three-times with PBST and mounted with Fluoroshield with DAPI (Sigma Aldrich) and stored at −20 °C until imaged. Similarly, lung cryo-sections were stained to determine PVX localization within the lung tissues with respect to integrin and macrophages.

For CD31 staining, tumor sections were permeabilized using 0.2% (v/v) Triton-X-100 (EMD Chemicals) in PBS for 2 min. Slides were blocked using 10% GS (v/v) in PBS and then incubated with A488-conjugated rabbit anti-mouse CD31 antibody (Biolegend) for 60 min, washed with PBS and then mounted as above and stored at −20 °C until imaged. Analysis of sections was performed on an Olympus Fluoview FV1000 confocal microscope. Images were processed using ImageJ software (http://imagej.nih.gov).

### Cell Uptake Using Flow Cytometry

HT-29 and RAW264.7 cells were grown to confluency in McCoy’s 5A media and DMEM media, respectively supplemented with fetal bovine serum (10% v/v) and penicillin/streptomycin (1% v/v) (all reagents from Life Technologies) at 37 °C and 5% CO_2_. Cells were washed three times with PBS and collected using enzyme-free Hank’s based Cell Dissociation Buffer (Fisher). Cells were then added to 96-well v-bottom plates (200,000 cells/200 *μ*L/well) and incubated with 1 *μ*g of RGD-PVX or PEG-PVX particles/well, in triplicate for 3 h at 37 °C and 5% CO_2_. Following incubation, cells were washed twice in FACS buffer [1 mM EDTA, 25 mM HEPES, 1% (v/v) FBS in PBS, pH 7.0] and fixed in 2% (v/v) paraformaldehyde (Electron Microscopy Sciences) in FACS buffer for 10 min at room temperature. Cells were washed twice after fixation, re-suspended in 400 *μ*L FACS buffer and stored at 4 °C until analysis. Cells were analyzed using a BD LSRII Flow Cytometer and 10,000-gated events were recorded. Data were analyzed using FlowJo 8.6.3 software.

For competitive binding assays, HT-29 cells were first incubated with 1,000-fold excess of free RGD peptide for 30 min followed by incubation with RGD-PVX particles for 2 h. For competitive binding assays with RAW264.7 cells a series of conditions was tested: RAW264.7 cells were pre-incubated with 10, 100, 1000 and 10,000× excess of soluble RGD peptide for 30 min followed by addition of RGD-PVX filaments, which were allowed to interact with the macrophage cell line for 2 h. Cells were then collected, prepared and analyzed as described above.

### Confocal Microscopy for Cellular Uptake

HT-29 cells and RAW264.7 cells were cultured as described above. For uptake studies, 25,000 HT-29 or RAW264.7 cells per well were cultured onto glass coverslips in a 24-well suspension culture plate for 24 h. After washing and replacing with fresh media, 1 *μ*g of RGD-PVX or PEG-PVX particles/well were added into the culture media and incubated with cells for 3 h. Post-incubation, cells were washed three times with sterile saline and fixed for 5 min at room temperature with DPBS containing 4% (v/v) paraformaldehyde and 0.3% (v/v) glutaraldehyde. Cells were then washed three times with DPBS. RAW264.7 cell coverslips were then mounted with Fluoroshield with DAPI (Sigma Aldrich) for nuclear staining and sealed using nail polish.

Post fixation, HT-29 cells were permeablized with 0.2% (v/v) Triton-X 100 (Fisher Bioscience) for 2 min followed by blocking with 10% (v/v) GS for 60 min. Cells were then stained with mouse anti-human LAMP-1 antibody (1:250 dilutions) (Biolegend) with 1% (v/v) GS in DPBS for 60 min, washed three times and then counter stained with A488-tagged goat anti-mouse antibody (at 1:1000 dilution, Life Technologies) with 1% (v/v) GS in DPBS for 60 min at room temperature. Coverslips were washed three times with DPBS, and mounted with Fluoroshield with DAPI (Sigma Aldrich) resulting in nuclear staining and sealed using nail polish. Confocal images were captured on Olympus FluoViewTM FV1000 LSCM and data processed using ImageJ 1.44o software (http://imagej.nih.gov/ij).

## Results and Discussion

### Synthesis of Targeted RGD-PVX and Stealth PEG-PVX Filaments

**Figure 2:**
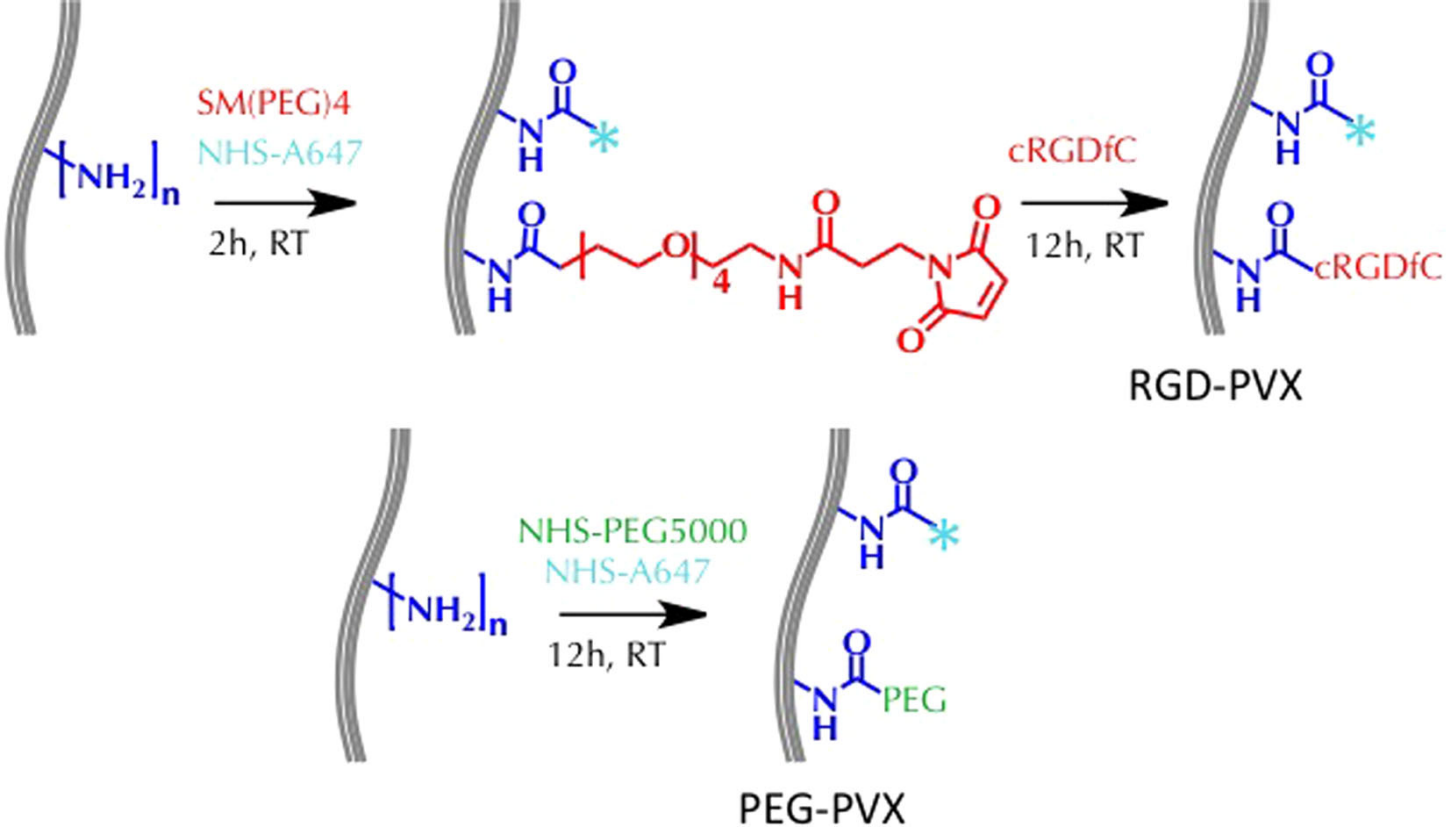
Modification of PVX with PEG and RGD ligands. *N*-hydroxysuccinimide (NHS) chemistry was used to conjugate A647 and SM(PEG)_4_ bifunctional linkers to PVX lysine residues. The cRGDfC peptide (with terminal cysteine) was then conjugated using the maleimide handle to produce RGD-PVX. Similarly, A647 and PEG (5-kDa MW) were conjugated to PVX by NHS chemistry to yield PEG-PVX filaments.

PVX was produced by molecular farming in *N. benthamiana* plants with yields of 20 mg pure PVX per 100 g infected leaf material. The RGD-PVX derivative was generated by conjugating the cyclic RGDfC peptide (with a terminal cysteine residue) to solvent exposed lysine residues on PVX using a bifunctional PEG linker with a 24.6 Å spacer arm [SM(PEG)_4_]. The PEGylated stealth formulation was prepared using linear PEG with a molecular weight of 5 kDa because PVX modified with this PEG had previously achieved tumor homing in various animal models.^31^^,^^32^ Filament tracking in cells and tissues was achieved by conjugating the near-infrared dye Alexa Fluor 647 succinimidyl ester (NHS-A647) using the reaction scheme outlined in Fig. 2.

**Figure 3:**
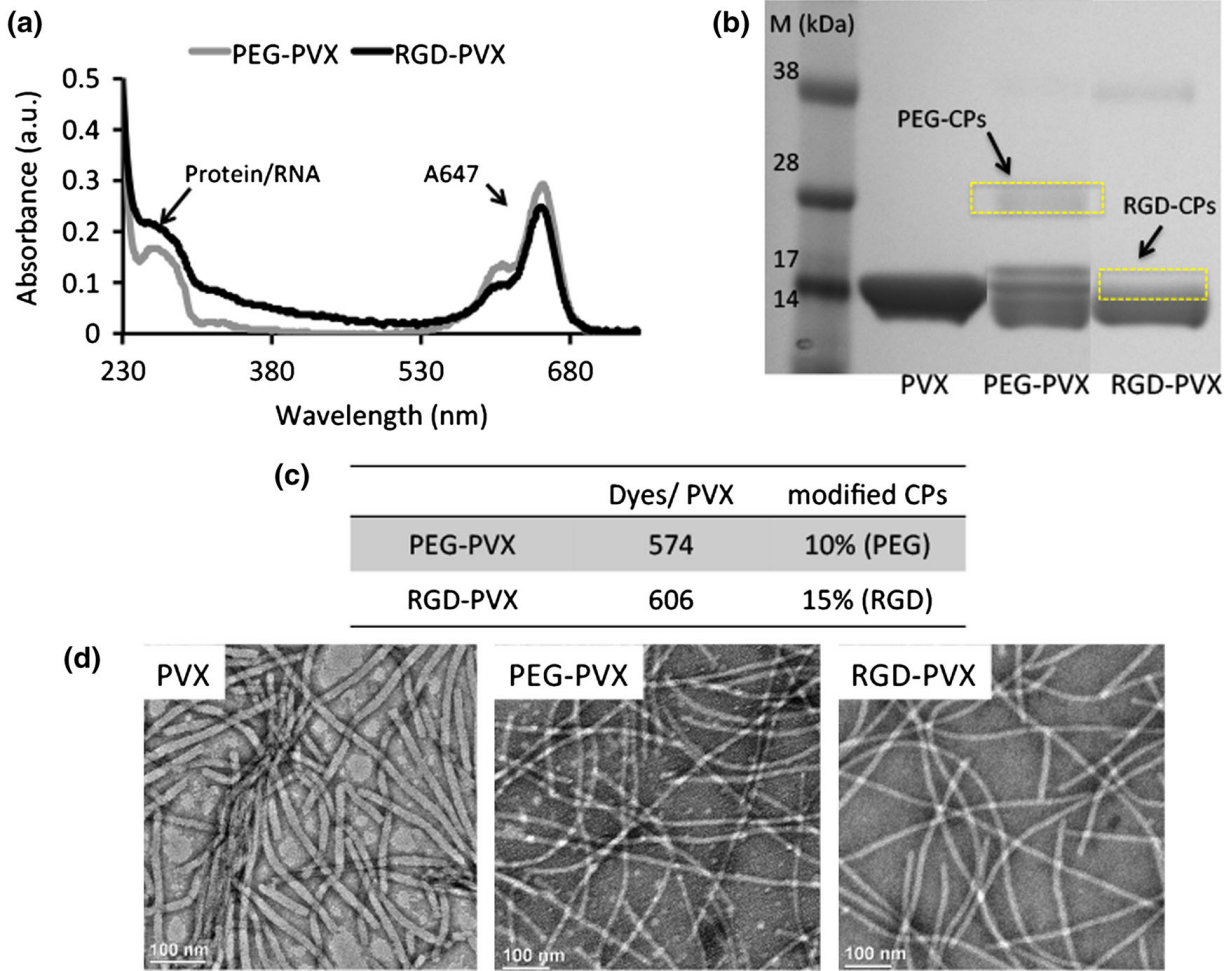
Characterization of PEG-PVX and RGD-PVX particles: (**a**) UV/Vis spectroscopy was used to determine number of dye molecules attached and to determine the concentration of PEG-PVX and RGD-PVX particles. (**b**) SDS-PAGE was used to confirm the covalent conjugation of PEG and RGD peptides to the PVX coat proteins (CPs). ImageJ software was used to determine the percentage of modified coat proteins. (**c**) Quantification of dye molecules and PEG/RGD ligands per PVX filament based on UV/Vis and SDS-PAGE. (**d**) Transmission electron microscopy was used to confirm the structural stability of PVX particles after modification (left to right: PVX, PEG-PVX and RGD-PVX). Scale bar = 100 nm.

The PVX-based formulations were characterized by UV/Vis spectroscopy and denaturing gel electrophoresis (SDS-PAGE) to confirm the degree of modification. Based on the Beer-Lambert law and the extinction coefficients of the fluorophore and PVX, approximately 574 dye molecules were conjugated per PEG-PVX and approximately 606 per RGD-PVX (Figs. 3a, 3c). SDS-PAGE confirmed the covalent attachment of the RGD peptide and PEG chains (Fig. 3b). The PVX coat protein has a molecular weight of 19 kDa, so higher molecular weights indicate the addition of RGD and PEG. ImageJ software was used to determine the degree of labeling showing that ~10% of the PVX coat proteins in the PEG-PVX formulation were modified with PEG5000, and ~15% of the PVX coat proteins were modified with RGD in the RGD-PVX formulation. The RGD-PVX particles therefore displayed ~200 RGD peptides by way of a 400-Da intervening PEG spacer, whereas the PEG-PVX particles displayed ~130 PEG chains with a molecular weight of 5 kDa.

### Biodistribution and Tumor Homing Properties

**Figure 4:**
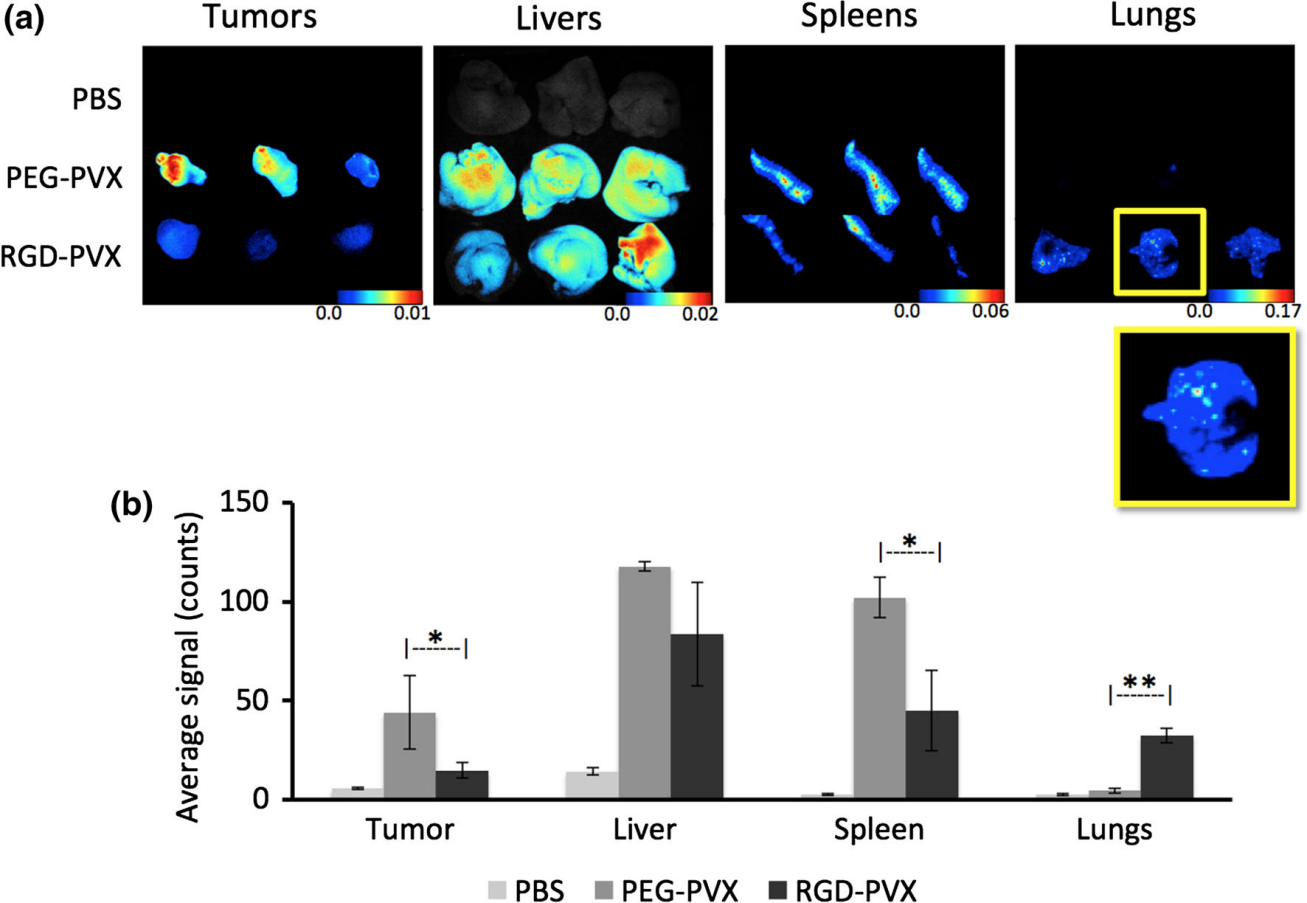
Comparative biodistribution of PEG-PVX and RGD-PVX particles injected intravenously into NCR-*nu/nu* mice with subcutaneous HT-29 tumor xenografts. *Ex vivo* Maestro analysis was carried out using dissected tissues 24 h post-administration. (**a**) Maestro images (yellow boxes show enlarged view of the lungs from animals treated with RGD-PVX, showing hot spots for PVX sequestration). (**b**) Quantitative analysis.

The biodistribution and tumor homing of the PVX formulations were studied using NCr-*nu/nu* nude mice with subcutaneous HT-29 xenografts. The nanoparticle formulations were administered intravenously as a single dose of 10 mg protein/kg body weight. At 24 h post-administration, the mice were sacrificed and the tumors, livers, spleens and lungs were removed for *ex vivo* quantitative tissue analysis using the Maestro™ fluorescence imaging system (Fig. 4).

Both formulations were cleared by the liver and spleen as expected because these are the organs of the mononuclear phagocytic system (MPS), which has been shown to facilitate the clearance of several virus-based nanomaterials, including those based on *Cowpea mosaic virus* (CPMV), *Tobacco mosaic virus* (TMV) and bacteriophages Qβ and M13.^5^^,^^13^^,^^21^^,^^28^^,^^34^ The overall biodistribution of the PEG-PVX stealth filaments was consistent with our previous observations.^31^^,^^32^ Although the majority of the injected PEG-PVX particles were cleared by the liver and spleen, a substantial amount also accumulated in the tumor tissue. In stark contrast, the RGD-targeted PVX formulation showed negligible accumulation in the tumor but significantly enhanced uptake in the lungs, where no stealth filaments were found (Fig. 4).

Our central hypothesis was that the RGD-targeted formulation would show similar or better levels of tumor homing compared to the stealth particles and would be more efficiently localized in the tumor because targeting would promote uptake by integrin-positive tumor cells, as has been observed with other nanoparticle systems.^30^ However, our experiments showed the opposite outcome, with the stealth particles but not the targeted particles accumulating in the tumor. The ability of the RGD-targeted particles to bind selectively to integrin was confirmed *in vitro* (see below), therefore we were able to rule out the lack of target specificity and recognition as an explanation for these unexpected results.

We considered the possibility that the unanticipated biodistribution of the particles might be explained by differences in their pharmacokinetic profiles. As stated above, PEG-PVX was decorated with ~130 PEG molecules, each with a molecular weight of 5 kDa, whereas RGD-PEG was decorated with ~200 RGD molecules, each with a 400-Da intervening PEG spacer. Based on the available surface area of PVX (*A*_PVX_ = 21,033 nm^2^) and the Flory dimension of 5-kDa PEG (*R*_F_ = 5.9 nm),^9^ we estimated that the PEG chains are presented in a mushroom conformation with nearly 70% surface area coverage. In contrast, the Flory dimension of the shorter PEG spacer (*R*_F_ = 0.8 nm) achieves less than 10% surface coverage. The more effective stealth effect of PEG-PVX is likely to protect the particles from serum protein adsorption, thus evading the immune system more effectively and resulting in more efficient passive tumor accumulation. This is consistent with reports describing the prolonged circulation of PEGylated liposomal doxorubicin formulations, which promotes EPR-mediated tumor homing.^22^^,^^23^

We attempted to increase the molecular weight of the intervening PEG spacer in the RGD-PVX formulation to match the stealth formulation, but integrin recognition of RGD-modified PVX with an intervening PEG 5-kDa spacer was diminished (data not shown). The longer PEG chain in the mushroom conformation may block the interaction between the RGD ligand and integrin thus preventing molecular recognition.

**Figure 5:**
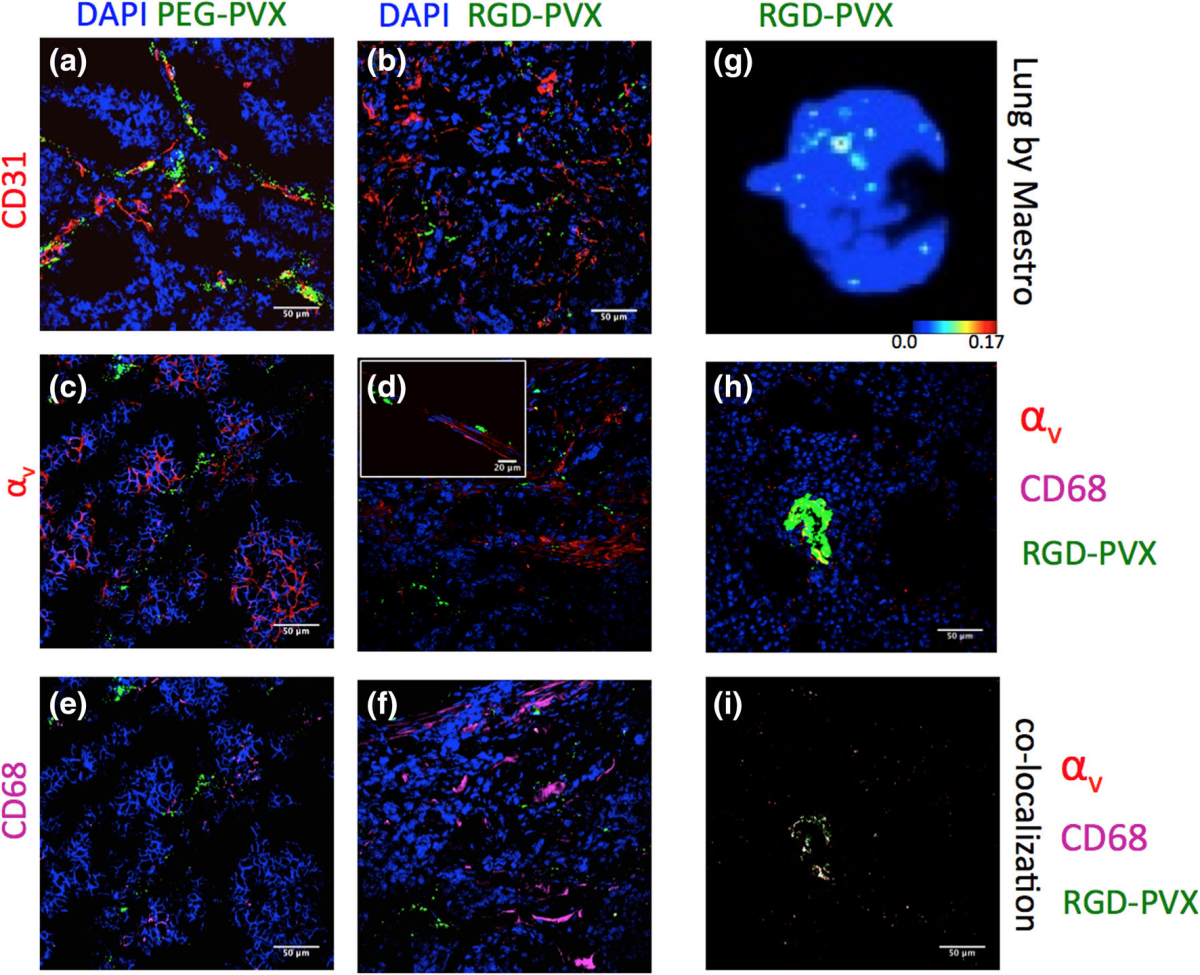
Immunofluorescence analysis of tumor and lung sections. The tumor sections were stained with antibodies specific for the endothelial marker CD31 (red, **a** + **b**), integrin *α*_v_ [red,** c** + **d** (inset: higher magnification)], the tumor macrophage marker CD68 (pink, **e** + **f**) and DAPI (blue) to determine the localization of PEG-PVX and RGD-PVX within the tumor. (**g**–**i**) Whole lung (**g**) and lung sections (**h**, **i**) from mice treated with RGD-PVX were stained for *α*_v_ integrin (red) and CD68^+^ macrophages (pink). Co-localization analysis was used to highlight the hotspots of RGD-PVX accumulation with integrins and CD68 macrophages (**i**). Scale bars = 50 *μ*m.

To gain further insight into the unexpected biodistribution profiles discussed above, we prepared tumor and lung sections from tumor xenograft mice and stained the sections using antibodies specific for the endothelium (CD31), *α*_v_ integrin and macrophages (CD68) as shown in Fig. 5. Histological analysis showed that both formulations were generally located near the tumor endothelium, but there was no significant co-localization with the endothelial marker CD31 (Figs. 5a, 5b). The tumor sections stained positive for *α*_v_ integrins as expected, since both HT-29 cells and the tumor endothelium overexpress *α*_v_ integrins,^11^ and the integrin-specific antibody we used cross-reacts with human *α*_v_ integrins (present on the cancer cells) and mouse *α*_v_ integrins (present on the endothelium). Although fewer RGD-PVX particles were found in the tumor tissue, they were associated with *α*_v_ integrin-positive sites within the tumor microenvironment (Fig. 5d). On the other hand, PEG-PVX stealth formulation did not colocalize with *α*_v_ integrins, which is in line with our expectations (Fig. 5c). Neither formulation appeared to colocalize with CD68-positive macrophages (Figs. 5e, 5f). The immunofluorescence data therefore confirmed that the stealth particles accumulated more efficiently than the targeted particles in the tumor. While both formulations were localized near the endothelium, the RGD-targeted particles were colocalized with *α*_v_ integrin-positive areas, indicating molecular specificity despite the diminished overall tumor accumulation.

Next, we investigated the histology of the lungs (Figs. 5g–5i). Only RGD-PVX was sequestered in the lungs, so tissue sections were prepared only from animals treated with the RGD-PVX formulation. Lung tissue sections were stained for *α*_v_ integrins and macrophages (using the CD68 marker). RGD-PVX was accumulated in hotspots in the lungs, and these areas were positive for both *α*_v_ integrins and CD68 (Figs. 5h, 5i).

**Figure 6:**
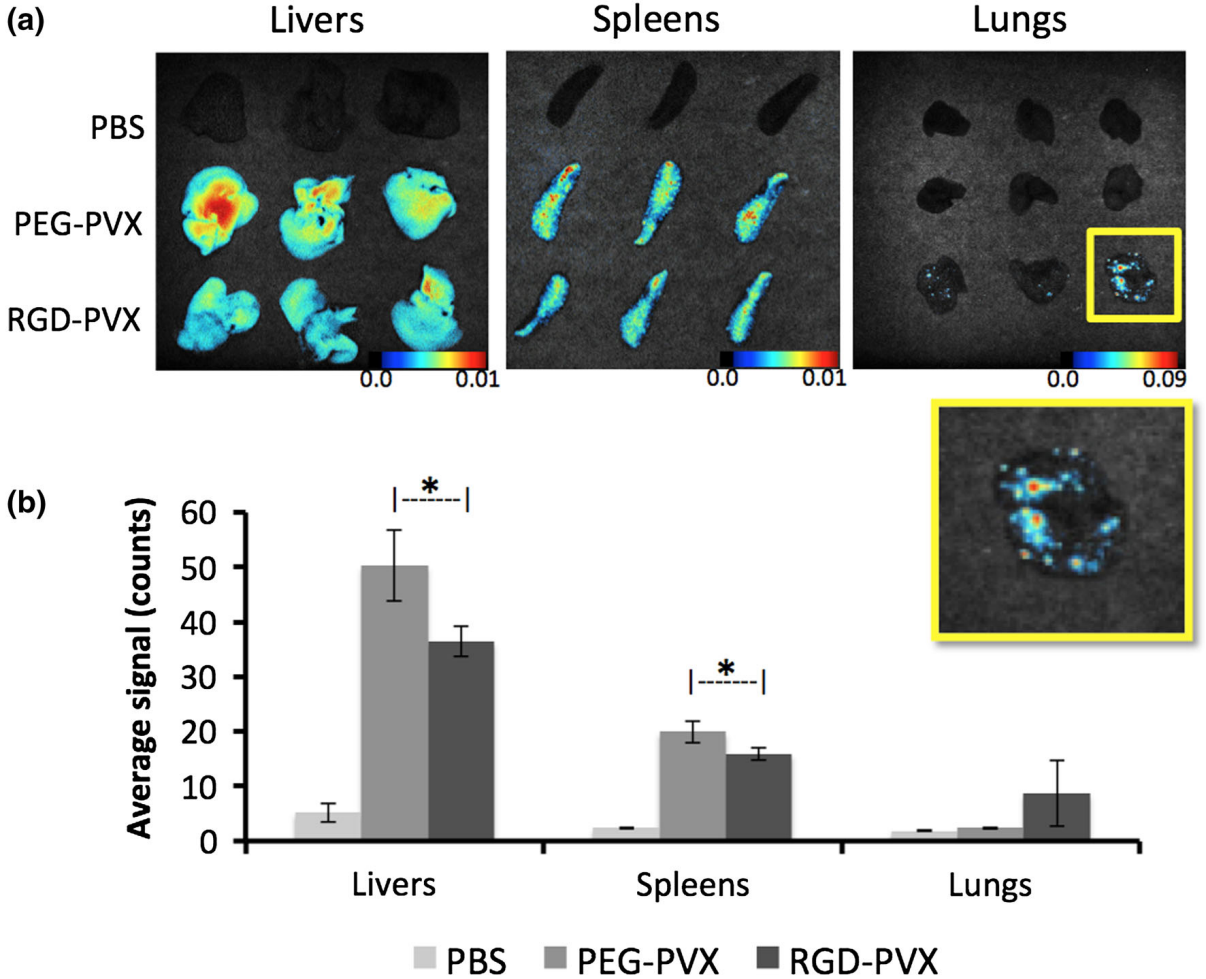
Biodistribution of PEG-PVX and RGD-PVX particles in healthy Balb/c mice. Tissues were collected for *ex vivo* Maestro™ fluorescence analysis 24 h after the administration of PEG-PVX and RGD-PVX. (**a**) Maestro images (yellow boxes show enlarged view of the lungs from animals treated with RGD-PVX, showing hot spots for PVX sequestration). (**b**) Quantitative analysis.

RGD-tagged nanoparticles have been reported to target lung metastasis 16^,^^25^ but the mouse model used in our experiments does not present metastatic disease in the lungs. This was confirmed by staining for human cells within the lungs using an anti-human vimentin antibody, and no human cells were found (data not shown). To ensure that the biodistribution profile was not specific to our tumor mouse model, we repeated the experiments using healthy Balb/c mice (Fig. 6). The results were similar to those obtained in the tumor xenograft models, i.e., both types of particles were cleared by the liver and spleen whereas only the RGD-PVX particles were sequestered in the lungs.

Our data indicate that targeted RGD-PVX filaments accumulate in the lungs in healthy animals as well as tumor xenograft models. The RGD-PVX filaments are sequestered in hotspots and are colocalized with *α*_v_ integrins and the macrophage marker CD68. Integrins play a role in macrophage-mediated inflammatory responses.^2^ Alveolar macrophages and the bronchiolar epithelia of normal lungs express integrins, but integrin expression is more extensive during inflammation.^33^ These background levels of integrin within the lung microenvironment may explain the sequestration of RGD-targeted nanoparticles in the lungs. Carbon nanotubes can also accumulate in the lungs^41^^,^^42^ but little information is available about other nanoparticles accumulating in the lungs following intravenous injection. A recent study showed prolonged accumulation of RGD-nanoparticles in the lungs following systemic administration.^1^

### Interactions with Cancer Cells and Macrophages

**Figure 7:**
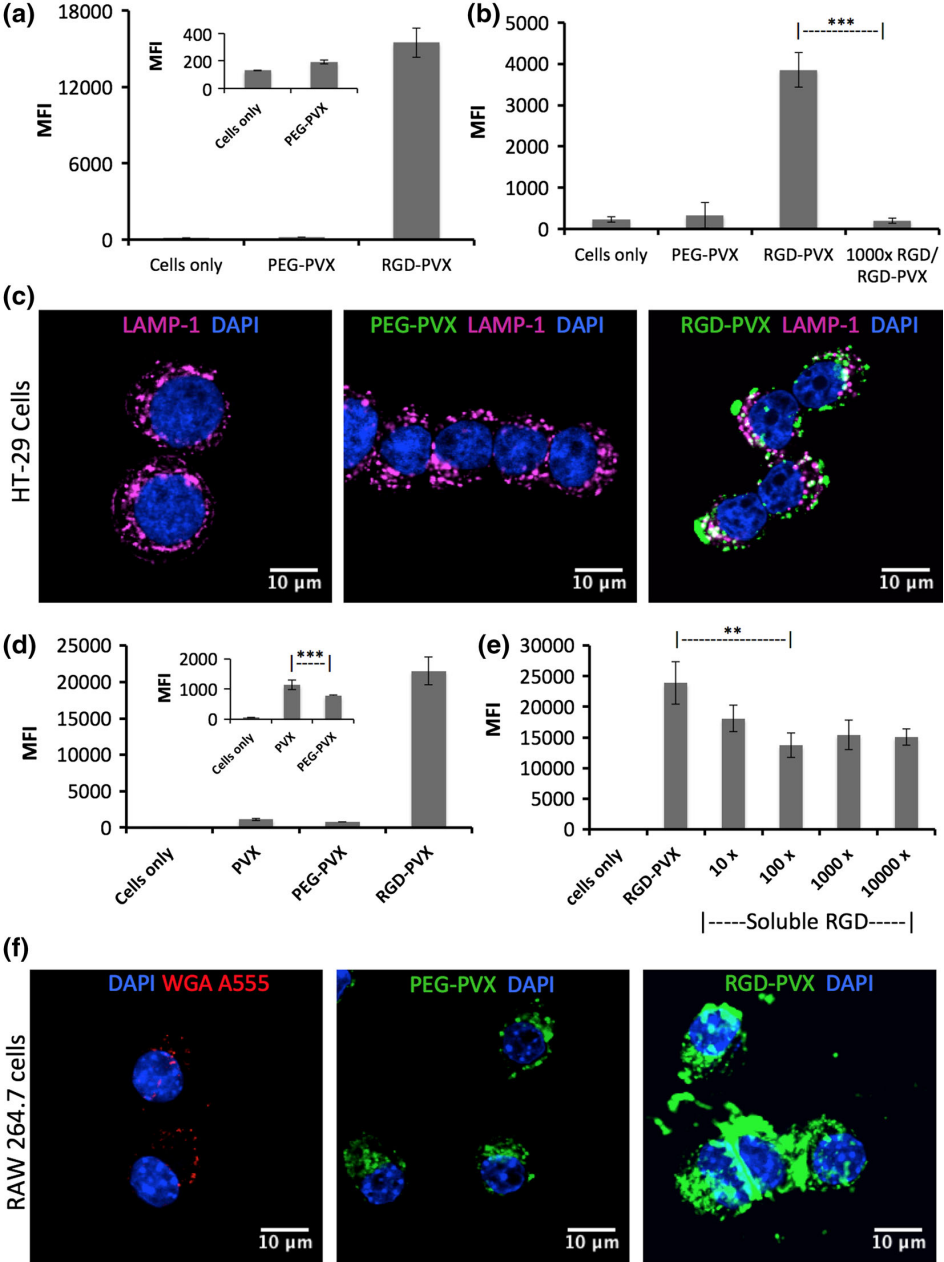
The analysis of PEG-PVX and RGD-PVX interactions with and uptake into cells using flow cytometry and confocal microscopy. (**a** + **d**) Flow cytometry was used to determine the interactions between PVX particles and HT-29 cancer cells (**a**, **b**) or RAW264.7 macrophages (**d**, **e**). The mean fluorescence intensity (MFI) is plotted and error bars indicate the standard deviation (*n* = 3). (**b** + **e**) Competition binding assays using RGD-PVX and free RGD peptides. (**c** + **f**) Confocal microscopy shows that RGD-PVX is taken up more efficiently than PEG-PVX by HT-29 cells and RAW264.7 macrophages. Cells were stained with DAPI (blue) to show the nucleus and with the endosomal marker LAMP-1 (pink; in HT-29 cells) or WGA (red; RAW cells). Colocalization of the particles and the endosomal marker indicates cellular uptake. Scale bars = 10 *μ*m.

To gain further insight into the behavior of the PVX filaments *in vivo*, we conducted a series of *in vitro* experiments using HT-29 cells (a colon carcinoma cell line) and RAW264.7 cells (a mouse macrophage cell line). RGD-targeted and stealth PVX particles were incubated with each of the cell lines and then analyzed by flow cytometry and confocal microscopy. PEG shielding minimizes the non-specific interaction between PVX particles and cells, so few particles were taken up by either cell line (Figs. 7a, 7d). In contrast, the RGD-targeted particles showed significant interactions with both the HT-29 and RAW264.7 cell lines (Figs. 7a, 7d) and were readily taken up into both cell types (Figs. 7c, 7f). Competition binding assays were carried out to investigate the molecular specificity of the RGD-targeted formulation. We found that a molar excess of 1000 free RGD peptides was able to prevent the molecular interaction between RGD-PVX particles and HT-29 cells, confirming that the RGD-PVX particles target integrins expressed on the surface of these cells (Fig. 7b).

Although stealth filaments were effectively shielded from phagocytosis, RGD-targeted filaments were also taken up by RAW264.7 cells (Figs. 7d–7f). PEG shielding reduces phagocytosis, but it is clear that the shorter PEG chain on the RGD-targeted formulation is unable to prevent non-specific clearance. To establish whether the RGD ligands also promote the uptake of RGD-PVX particles by the macrophage cell line, we repeated the competition binding assays, this time pre-incubating RAW264.7 cells with free RGD peptides at various molar excesses before adding the RGD-PVX particles. The presence of soluble RGD did not completely abolish the uptake of RGD-PVX particles but there was a significant reduction compared to the untreated control, suggesting that *α*_v_ integrin plays a role in this process (Fig. 7e). This is consistent with other studies showing that RGD-modified particles were taken up by monocytes. In contrast to our study, nanoparticle–monocyte targeting was a successful way to target the nanoparticles to the tumor tissue by promoting monocyte delivery.^35^

## Conclusions

We compared RGD-PVX and PEG-PVX filaments to determine their tumor homing properties based on biodistribution and cellular interaction *in vitro* and in tumor-bearing and healthy mice. The stealth PVX particles showed significant tumor homing capability, facilitated by their longevity in the circulation and therefore their prolonged bioavailability, as demonstrated by their slow phagocytic clearance. In contrast, RGD-targeted PVX filaments showed negligible tumor homing capability but were instead sequestered in the lungs. The lung-specific accumulation of the RGD-modified filaments probably reflects their ability to bind integrins expressed on alveolar macrophages and the bronchiolar epithelium.^33^ In future studies, one might consider utilizing the propensity of RGD-PVX filaments to target inflamed areas within the lung microenvironment to block integrin-mediated interactions with metastatic cells that facilitate tumor progression, but this would require further careful optimization.

Although molecular targeting may promote cellular uptake, the targeting ligand also affects the overall biodistribution of the carrier. We found that the addition of a targeting ligand may not necessarily achieve favorable tumor partitioning. Although RGD peptides are useful for preclinical and clinical investigation, their incorporation into PVX-based stealth filaments was only successful *in vitro*. Alternative design strategies, e.g., incorporation of the targeting ligands into more heavily PEGylated filaments, or the targeting of alternative molecular receptors such as growth factors that are up-regulated or selectively expressed within the tumor microenvironment, may be suitable strategies to achieve molecular targeting of the filamentous carrier. Overall, these studies provide an important insight into the design principles that are required for virus-based nanoparticles to achieve selective delivery of diagnostic or therapeutic cargos to tumor cells.
